# First Trimester Vitamin D Deficiency and Risk of Gestational Diabetes Mellitus in a Mexican Cohort

**DOI:** 10.3390/nu18010097

**Published:** 2025-12-27

**Authors:** Lidia Arce-Sánchez, Isabel González-Ludlow, Ileana Lizano-Jubert, Jocelyn Andrea Almada-Balderrama, Blanca Vianey Suárez-Rico, Araceli Montoya-Estrada, Guadalupe Estrada-Gutierrez, Maribel Sánchez-Martinez, Juan Mario Solis-Paredes, Johnatan Torres-Torres, Ameyalli Mariana Rodríguez-Cano, Maricruz Tolentino-Dolores, Otilia Perichart-Perera, Mariana Villegas-Soto, Enrique Reyes-Muñoz

**Affiliations:** 1 Coordination of Endocrinology, Instituto Nacional de Perinatología Isidro Espinosa de los Reyes, Mexico City 11000, Mexico; li_arce@yahoo.com.mx (L.A.-S.); jocelyn.almada@gmail.com (J.A.A.-B.); 2 Nutrition and Bioprogramming Coordination, Instituto Nacional de Perinatología Isidro Espinosa de los Reyes, Mexico City 11000, Mexicorocameyalli@gmail.com (A.M.R.-C.); cruz_tolentino@yahoo.com.mx (M.T.-D.); otiliaperichart@inper.gob.mx (O.P.-P.); 3 Coordination of Gynecological and Perinatal Endocrinology, Instituto Nacional de Perinatología Isidro Espinosa de los Reyes, Mexico City 11000, Mexico; ileana1500@gmail.com; 4 Facultad de Ciencias de la Salud, Universidad Anáhuac México, Campus Norte, Huixquilucan 52786, Mexico; 5 Department of Immunobiochemistry, Instituto Nacional de Perinatología Isidro Espinosa de los Reyes, Mexico City 11000, Mexico; blancasuarezrico@gmail.com (B.V.S.-R.); guadalupe.estrada@inper.gob.mx (G.E.-G.); maribel1971sm@gmail.com (M.S.-M.); maliviso99@gmail.com (M.V.-S.); 6 Department of Reproductive and Perinatal Health Research, Instituto Nacional de Perinatología Isidro Espinosa de los Reyes, Mexico City 11000, Mexico; juan.mario.sp@gmail.com (J.M.S.-P.); johnatan.torres@inper.gob.mx (J.T.-T.); 7 Facultad Interdiscliplinaria de Ciencias Biológicas y de Salud, Universidad de Sonora, Campus Hermosillo, Hermosillo 83000, Mexico

**Keywords:** vitamin D deficiency, gestational diabetes mellitus, adverse perinatal outcomes

## Abstract

**Background/Objectives:** Vitamin D deficiency has been associated with an increased risk of adverse perinatal outcomes (APOs). This study aimed to examine whether vitamin D deficiency during the first trimester of pregnancy is linked to the development of gestational diabetes mellitus (GDM) in a Mexican population. **Methods:** A total of 404 pregnant women from the Biochemical and Epigenetic Origin of Overweight and Obesity (OBESO) cohort were included. Maternal vitamin D levels were measured between 11 and 14 weeks of gestation. Vitamin D deficiency was defined as a level below 20.0 ng/mL. The primary goal was to compare APOs between Group 1 (women with vitamin D deficiency) and Group 2 (women without vitamin D deficiency). Adjusted odds ratio (aOR) for APOs—including GDM, preeclampsia, preterm birth, miscarriage, cesarean section, and neonatal size—were calculated, adjusting for pregestational body mass index (BMI) and obesity, with 95% confidence interval (95% CI). **Results:** Vitamin D deficiency was present in 40.5% of women. Pre-pregnancy BMI and obesity were significantly higher in women with deficiency; other baseline characteristics did not differ between groups. Women with vitamin D deficiency had a higher risk of GDM (aOR 2.04, 95% CI 1.14–3.65, *p* = 0.01). No association was found between vitamin D deficiency and other APOs. **Conclusions:** The incidence of vitamin D deficiency in the first trimester was 40.5%. Early pregnancy vitamin D deficiency increases the risk of GDM among Mexican women. These findings highlight the importance of monitoring and supplementing vitamin D during pregnancy to reduce the risk of GDM.

## 1. Introduction

Vitamin D is a fat-soluble steroid prohormone with endocrine, autocrine, and paracrine functions; it is mainly recognized for maintaining calcium and phosphorus homeostasis [1,2,3,4]. Besides its role in bone metabolism, its anti-inflammatory and immunomodulatory effects have also been demonstrated [4]. Conversely, vitamin D insufficiency (between 20 and 30 ng/mL) and deficiency (below 20 ng/mL) have been associated with various extra-skeletal adverse effects, including chronic diseases, immune system dysfunction, a heightened risk of cardiovascular disease, and changes in neuropsychiatric functions, among others [5,6,7]. 

Although vitamin D is obtained through exposure to ultraviolet (UV) radiation and dietary intake [5], its deficiency has increased due to modern lifestyle practices such as sun protection, cosmetic products, limited sunlight exposure, smoking, obesity, inadequate vitamin D intake, intestinal malabsorption, seasonal changes, conditions like kidney or liver failure, chronic inflammation, and medication use [4].

It is estimated that, worldwide, about 54% of pregnant women experience vitamin D insufficiency, and 18% have a deficiency [8]. In Mexico, according to the 2023 National Health and Nutrition Survey (ENSANUT), the prevalence of vitamin D insufficiency and deficiency among women of reproductive age was 46.6% and 43.8%, respectively [9].

Evidence links vitamin D deficiency to a higher risk of fetal problems during pregnancy and in the neonatal period, such as fetal growth restriction, preterm birth, low birth weight, anthropometric abnormalities, neurodevelopmental disorders, and respiratory infections [10,11]. 

Maternal associations described in the literature include increased risk of miscarriage, vaginal infections, placental insufficiency, preeclampsia, C-section, postpartum depression, and autoimmune thyroid diseases [12,13,14,15,16,17,18]. A recent systematic review of 29 observational studies concluded that there is a link between vitamin D deficiency and higher risk of gestational diabetes mellitus (GDM) (OR: 1.26; 95% CI: 1.13–1.41). However, the review noted significant heterogeneity across studies (*I*^2^ = 71%), and no research was conducted in Latin American populations [17].

The International Diabetes Federation (IDF) reported that diabetes during pregnancy affected 23 million women worldwide in 2024 [19]. In Mexico, approximately 14.6 million people had diabetes in 2022 [20], and according to IDF in 2024, the pregnancy diabetes prevalence in North America and the Caribbean region was 22.4%, with 80% attributed to GDM [19].

Various risk factors have been identified for the development of GDM, including personal and family history of the disease, signs of insulin resistance such as acanthosis nigricans, pregestational overweight or obesity, polycystic ovary syndrome, cardiovascular disease, and advanced maternal age, among others [21,22]. However, its etiology remains unclear.

Some studies have examined the link between vitamin D and blood glucose levels, but the results have been mixed. Evidence suggests that vitamin D might influence glucose regulation by binding to pancreatic β-cell receptors, producing 1α-hydroxylase enzyme, and regulating extracellular calcium, which affects insulin sensitivity and secretion. However, its direct connection to GDM has not yet been proven [4].

Although some studies have examined the relationship between vitamin D and blood glucose levels, results remain inconsistent. Emerging evidence indicates that vitamin D might affect glucose regulation by binding to vitamin D receptors in pancreatic β-cells, allowing local activation through the 1α-hydroxylase enzyme, and controlling extracellular calcium—mechanisms that are crucial for insulin secretion and sensitivity [3]. However, a direct causal relationship between vitamin D levels and the development of gestational diabetes mellitus (GDM) has not yet been conclusively proven [3].

Given the increasing recognition of hypovitaminosis D in pregnancy as a major global health concern, along with growing evidence linking it to adverse maternal outcomes, GDM has gained attention as a potential early marker of glucose intolerance and future type 2 diabetes.

This study aimed to determine the association between vitamin D deficiency during the first trimester of pregnancy and the development of GDM in a Mexican population.

## 2. Materials and Methods

### 2.1. Study Design and Participants 

This was a prospective cohort study involving pregnant women enrolled in the Biochemical and Epigenetic Origins of Overweight and Obesity (OBESO) cohort at the National Institute of Perinatology (INPER) in Mexico City. The protocol was approved by the Research and Ethics Committee of INPerIER in Mexico City (protocol number 33300-11-402-01-575-17; continuation, register number 2024-1-14). All participants provided written informed consent.

The inclusion criteria consisted of women who initiated prenatal care during the first trimester of pregnancy between 2017 and 2022, with a vitamin D measurement performed between gestational weeks 11 and 14.

Exclusion criteria included women with comorbid conditions such as pre-gestational diabetes mellitus, systemic arterial hypertension, as well as cardiovascular, renal, immunological, neurological, hematological, oncological diseases, or HIV infection. Additional exclusion criteria were incomplete clinical records and pregnancies not resolved at the Institute.

### 2.2. Procedure

All women who participated in the study provided a blood sample between 11 and 14 weeks of gestation after fasting for 8 to 10 h. Blood samples were centrifuged at 5000 revolutions per minute for 10 min, and the serum was stored at −70 °C until analysis. The serum concentration of 25-hydroxyvitamin D (25-OH-D) was measured using a chemiluminescence immunoassay performed on the Architect system (Abbott Diagnostics, Lake Forest, IL, USA) [23]. Vitamin D deficiency was defined as a serum vitamin D level of <20 ng/mL [4].

All participants received prenatal care from the departments of obstetrics and maternal-fetal medicine, with consultations every 4 weeks until 32 weeks of gestation. Subsequently, follow-ups were conducted every 2 weeks until resolution, in accordance with institutional protocol. Demographic and clinical data from admission to prenatal care to delivery were collected from clinical records.

### 2.3. Study Variables

The primary outcome was to determine the risk of GDM in Mexican women with vitamin D deficiency. GDM was defined as the presence of one or more abnormal values during the 2 h, 75 g oral glucose tolerance test (OGTT), diagnosed for the first time between 24 and 28 weeks of pregnancy, according to the World Health Organization (WHO) cut-off points [19,24]: Fasting glucose ≥ 92 mg/dL, 1 h glucose ≥ 180 mg/dL, and 2 h glucose ≥ 153 mg/dL. The OGTT was performed for all participants as part of the prenatal care protocol at the INPER.

The secondary outcomes were the following adverse perinatal outcomes (APOs): (1) Preeclampsia: defined as systolic blood pressure ≥ 140 mmHg and/or diastolic blood pressure ≥ 90 mmHg in a previously normotensive woman, after 20 weeks of gestation, accompanied by proteinuria and/or severe features [25]. (2) Preterm birth: defined as any delivery between 20 and 36.6 weeks of gestation [26]. (3) Neonate born small for gestational age: defined as birth weight below the 10th percentile for gestational age and sex, based on Mexican population references [27]. (4) Miscarriage, defined as the natural ending of a pregnancy before 20 weeks of gestation or when the fetal weight is less than 500 g.

Insulin resistance in the first trimester was defined as Homeostatic Model Assessment of Insulin Resistance (HOMA_IR) higher than 1.6 [28]. Neonatal records were examined for gestational age at birth, weight, height, Apgar scores, and complications.

### 2.4. Sample Size

To detect a difference in the incidence of GDM between women with vitamin D deficiency (20%) and those without deficiency (10%), a sample size calculation for proportions was conducted. Assuming a 95% confidence level, α = 0.05, and 80% power, at least 144 participants per group were required.

### 2.5. Statistical Analysis

Continuous variables were expressed as means and standard deviations, while categorical variables were reported as frequencies and percentages. Depending on the distribution, comparisons between continuous variables were performed using Student’s *t*-test or the Mann–Whitney U test. For categorical variables, chi-square tests or Fisher’s exact tests were used as appropriate. A *p*-value ≤ 0.05 was considered statistically significant. Subsequently, a contingency table was used to obtain odds ratios (ORs) and 95% CIs for each variable associated with vitamin D deficiency. Finally, a logistic regression analysis using the forward method was performed to assess APOs adjusted for body mass index and obesity, reporting adjusted odds ratios (aORs) with 95% CIs. Statistical analyses were conducted using SPSS software, version 24 (IBM Corp., Armonk, NY, USA).

### 2.6. Use of Generative Artificial Intelligence Tools

During the writing of this manuscript, the authors used Grammarly [https://www.grammarly.com], San Francisco, CA, USA (URL accessed on 5 November 2025), and ChatGPT (version GPT-5, OpenAI, San Francisco, CA, USA) for grammar and editing revisions. The authors have reviewed and edited the output and assume full responsibility for the content of this publication.

## 3. Results

**Figure 1:**
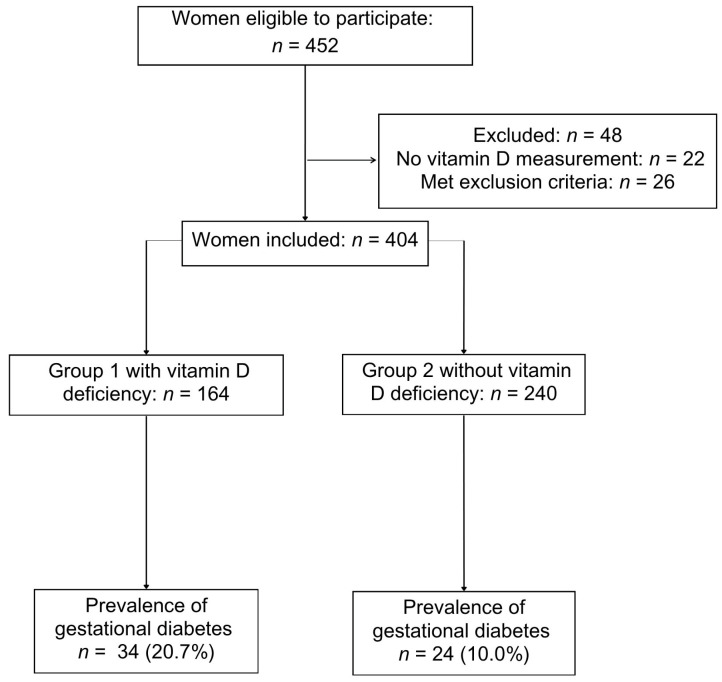
Flowchart of study participants.

Out of 452 women eligible to participate, a total of 404 women were included. Figure 1 shows the flowchart of participants in the study. Women who met the inclusion criteria were enrolled, with 164 in Group 1 (vitamin D deficiency) and 240 in Group 2 (without deficiency). 

Table 1 presents the baseline characteristics of participants at enrollment for prenatal care. There were no significant differences between groups regarding maternal age, educational level, marital status, insulin resistance, pregestational body mass index (BMI), aspirin use, pregestational weight, parity, physical activity, or history of preterm birth.

Prepregnancy BMI and the percentage of women with obesity were significantly higher in the vitamin D deficiency group. A notably higher rate of vitamin D deficiency was observed among women enrolled in autumn and winter compared to those in spring and summer (*p* = 0.025).

As expected, the average vitamin D concentration was significantly lower in those with vitamin D deficiency (*p* = 0.0001).

Table 2 shows the characteristics of women during pregnancy and their newborns in the study groups. There were no differences in gestational age at delivery, newborn weight, gestational age at OGTT, fasting glucose during OGTT, women taking aspirin started before 17 weeks of gestation, or women beginning metformin during the first or second trimester of pregnancy. The use of metformin was indicated by the obstetrician during prenatal care; most women who take metformin during the first trimester have obesity and a history of miscarriage. During the second trimester, use of metformin was mainly due to gestational diabetes. The percentage of women taking vitamin D supplements starting in the first or second trimester was similar in both groups—40.9% in the vitamin D deficiency group and 43.8% in the no deficiency group (*p* = 0.563). Most women taking multivitamins used a dose containing an interquartile range of 250 to 500 IU of cholecalciferol.

The risk of APOs, adjusted for pregestational BMI and obesity, expressed as aOR in women with and without vitamin D deficiency, is shown in Table 3. There were no significant differences in miscarriage, preterm birth, preeclampsia, gestational hypertension, cesarean delivery, or neonates large or small for gestational age. 

Vitamin D deficiency was an independent risk factor that increased the risk of GDM, with an aOR of 2.04 (95% CI 1.14–3.65), *p* = 0.01. The overall prevalence of GDM was 14.4%. Of the 58 GDM cases, 46.5% (*n* = 27) occurred during autumn and winter, compared to 53.5%% (*n* = 31) in spring and summer (*p* = 0.57). 

## 4. Discussion

### 4.1. Principal Findings

In this prospective cohort of pregnant women from Mexico, we found that first-trimester vitamin D deficiency was highly prevalent (40.5%) and independently associated with an increased risk of developing GDM. Women with vitamin D deficiency had nearly twice the risk of GDM, with an aOR of 2.04 (95% CI 1.14–3.65), *p* = 0.01, compared to women with sufficient vitamin D levels. No significant connections were observed between vitamin D deficiency and other adverse pregnancy outcomes, including miscarriage, preterm birth, preeclampsia, cesarean, gestational age at birth, neonatal birth weight, small-for-gestational-age or large-for-gestational-age infants.

These findings support the hypothesis that maternal vitamin D deficiency during early pregnancy may contribute to glucose dysregulation and the development of GDM, while its impact on other perinatal complications remains less clear.

### 4.2. Comparison with Existing Literature

Our results are consistent with previous observational studies and meta-analyses indicating that low maternal vitamin D levels are associated with an increased risk of developing GDM. A case–control study involving 100 women found significantly lower vitamin D levels in those with GDM compared to normoglycemic controls (OR = 1.83; *p* = 0.010), despite using a different deficiency cutoff (<25 ng/mL) [29]. Similarly, a meta-analysis of 26 studies showed that women with vitamin D deficiency faced a small but statistically significant higher risk of GDM (pooled OR = 1.18; 95% CI: 1.01–1.35; *p* < 0.001) [17]. 

Recent research has also explored potential factors that influence this relationship, particularly maternal BMI and insulin resistance. Some studies have shown stronger associations among women with obesity or metabolic syndrome, suggesting combined effects on glucose metabolism [30]. However, in our population, the link between vitamin D deficiency and GDM persisted after adjusting for BMI, and no significant interaction was observed.

Additionally, this study observed fewer GDM cases during autumn and winter, accounting for 46.5% of the total cases, compared to 53.5% in spring and summer. However, this difference was not statistically significant, likely due to the sample size, unlike the findings of Chiefari et al., who reported a significantly higher GDM incidence in summer and a decrease during the colder months [31]. This could partly be explained by higher vitamin D deficiency during the first trimester of pregnancy in autumn and winter, and an increased incidence of GDM in the late second trimester (weeks 24–28) during spring and summer. 

On the other hand, several studies conducted in low-risk or homogeneous populations have not demonstrated a clear connection between vitamin D levels and GDM [32]. This variation across studies may result from differences in when vitamin D was measured, ethnic background, sunlight exposure, dietary intake, and the methods used to measure 25(OH)D.

In line with other cohorts, we did not find links between vitamin D deficiency and maternal or neonatal outcomes such as preterm birth, preeclampsia, or low Apgar scores [33]. This suggests that the impact of vitamin D deficiency may mainly concern glucose regulation during pregnancy rather than a broader range of obstetric issues, at least in the first trimester.

### 4.3. Potential Biological Mechanisms

Multiple lines of experimental and clinical evidence support a biologically plausible link between vitamin D status and glucose homeostasis relevant to GDM. During pregnancy, calcitriol (1,25-(OH)2D3) binds the vitamin D receptor expressed in pancreatic β-cells and insulin-responsive tissues; β-cells and the placenta express CYP27B1, enabling local activation of vitamin D and autocrine/paracrine signaling [34]. In β-cells, (1,25-(OH)2D3)/vitamin D receptor complexes regulate insulin gene transcription via vitamin D response elements and modulate intracellular Ca^2+^ handling, which is essential for glucose-stimulated insulin exocytosis—defects that are reversible in deficiency models with calcitriol [35]. Beyond β-cell secretion, vitamin D can enhance peripheral insulin action by influencing insulin receptor/signaling pathways and GLUT4-mediated glucose uptake, while also exerting anti-inflammatory effects (e.g., attenuation of NF-κB–dependent cytokines) that counteract the low-grade inflammatory milieu contributing to gestational insulin resistance [36]. Consistent with these mechanisms, several prospective pregnancy cohorts have shown that lower first-trimester vitamin D levels are associated with higher insulin resistance later in pregnancy and a greater risk of GDM; however, variability across studies remains [37]. Our findings in the OBESO cohort align with this evidence; women with early-pregnancy vitamin D deficiency had nearly a two-fold higher risk of developing GDM compared to those with sufficient levels, supporting the idea that inadequate vitamin D availability during early gestation may worsen the insulin resistance typical of pregnancy and contribute to dysglycemia.

Aggregated evidence from meta-analyses also shows that low or deficient vitamin D levels are associated with higher odds of GDM, although intervention data are mixed and underscore the need for well-powered, early-pregnancy trials focusing on women with documented deficiency [38]. Overall, these pathways—β-cell dysfunction, reduced insulin sensitivity, and increased inflammatory signaling—offer a comprehensive framework for understanding how early-pregnancy hypovitaminosis D could contribute to the development of GDM [34].

### 4.4. Strengths and Limitations

The main strength of our study is its prospective design, featuring standardized first-trimester measurement of 25(OH)D using a validated immunoassay, which allows for temporal inference and minimizes reverse causation. The study was carried out in a well-characterized Mexican cohort, providing valuable data from a population often underrepresented in the global literature. Additionally, the thorough assessment of pregnancy outcomes and adjustment for key confounders (BMI, parity, insulin resistance, seasonality, and physical activity) strengthen the reliability of the findings.

Nonetheless, several limitations must be acknowledged. The study was conducted at only one tertiary referral center, which may limit its relevance to other regions or healthcare settings. Data on sunlight exposure, dietary intake, and vitamin D supplementation was incomplete, preventing a thorough analysis of these factors. Vitamin D was measured only once during early pregnancy; thus, we could not assess changes over time or in the third trimester. Finally, although our models adjusted for key confounders, residual confounding and measurement variability cannot be entirely eliminated.

Future research should involve multicenter cohorts and randomized controlled trials to determine causality, identify optimal vitamin D levels for metabolic health, and evaluate whether supplementation during early pregnancy can effectively prevent GDM and improve perinatal outcomes.

### 4.5. Clinical Implications

From a clinical and public health perspective, our findings emphasize the importance of early vitamin D screening as part of comprehensive prenatal care, especially in populations with high rates of hypovitaminosis D and rising GDM prevalence. In Mexico, routine vitamin D testing is not yet a standard part of prenatal screening. Adding this assessment could allow early identification of women at higher metabolic risk and facilitate personalized supplementation strategies.

Considering the biological plausibility linking vitamin D to pancreatic β-cell function, insulin sensitivity, and inflammatory regulation, addressing maternal deficiency during the first trimester could be a practical and cost-effective preventive strategy. We support developing national and regional policies aimed at maintaining adequate maternal vitamin D levels through nutritional guidance, supplementation, and population-based prevention programs.

The definition of what constitutes “normality” levels and vitamin D deficiency is a heavily debated topic. While there is a consensus in the general population on the threshold of 10–12 ng/mL to define a condition of “vitamin D deficiency” (linked to rickets, osteomalacia, and secondary hyperparathyroidism) [39], the definition of “sufficiency” values in the general population remains controversial, the Italian Society for Osteoporosis, Mineral Metabolism and Bone Diseases suggested that, in the general population, including healthy elderly individuals, a threshold value of 25(OH)D ≥ 20 ng/mL (50 nmol/L) should be considered adequate and not require any supplementation [39].

Most experts agree that vitamin D deficiency should be defined as a 25(OH)D level below 20 ng/mL, with vitamin D supplementation recommended [40]. Meanwhile, a recent Endocrine Society guideline, based on the lack of supportive clinical trial evidence, recommends against routine 25(OH)D testing in the absence of specific indications and suggests empiric vitamin D for individuals aged 1 to 18 years, those over 75 years old, pregnant women, and individuals with high-risk prediabetes [41].

During pregnancy, many studies have reported links between 25(OH)D levels below 20 ng/mL (<50 nmol/L) and adverse perinatal outcomes [41,42].

Although 40.5% of women in the present study took vitamin D supplements, mainly from multivitamins, the percentages and doses were similar across groups and did not impact GDM incidence. This highlights another area for clinical research to identify the optimal vitamin D supplement doses for preventing GDM. The optimal dosage of vitamin D for preventing maternal and fetal complications remains unclear [41].

Finally, multicenter interventional studies are necessary to assess the clinical benefits of treating vitamin D deficiency on maternal glycemic outcomes and fetal growth, as well as to develop evidence-based guidelines tailored to specific contexts for prenatal care across Mexico and Latin America.

## 5. Conclusions

The incidence of vitamin D deficiency in Mexican women during their first trimester of pregnancy is 40.5%. Vitamin D deficiency at this stage is linked to an increased risk of developing GDM. The limited evidence available in Latin American populations, along with the current findings, emphasizes the importance of early screening for vitamin D levels in pregnant women. It also highlights the potential of supplementation strategies as a preventive measure to reduce GDM cases.

## Figures and Tables

**Table 1:** Characteristics of pregnant women with and without vitamin D deficiency at the time of prenatal care enrollment.

Characteristic	Vitamin D Deficiency (*n* = 164)	No Vitamin D Deficiency (*n* = 240)	*p*-Value
Age (years)	30.2 ± 5.5	29.9 ± 5.3	0.579
Pre-pregnancy BMI (kg/m^2^)	28.53 ± 5.90	26.74 ± 4.93	0.002
Fasting glucose (mg/dL)	75.8 ± 20	74.7 ± 21	0.980
Insulin (mcUI/mL)	14.2 ± 18.3	14.4 ± 2.4	0.898
HOMA-IR Index	2.09 ± 2.39	1.95 ± 2.46	0.586
Insulin resistance (HOMA-IR > 1.6)	69 (42.07%)	89 (37.08%)	0.419
Vitamin D concentration (ng/mL)	15.9 ± 3.23	26.24 ± 4.76	0.0001
Normal weight (BMI 18.5–24.99 kg/m^2^)	55 (33.5%)	97 (41.6%)	0.195
Overweight (BMI 25–29.99 kg/m^2^)	52 (31.7%)	80 (34.3%)	0.815
Obesity (BMI ≥ 30 kg/m^2^)	57 (34.8%)	56 (24.0%)	0.016
Primigravida	98 (59.8%)	136 (56.7%)	0.607
Multigravida	66 (40.2%)	104 (43.3%)	0.607
Autumn-Winter	91 (55.5%)	106 (44.2)	0.025
Spring-Summer	73 (44.5%)	134 (55.8%)	0.025

BMI = Body mass index; HOMA-IR = Homeostasis Model Assessment of Insulin Resistance; IT = first trimester.

**Table 2:** Characteristics of pregnant women and newborns in groups with and without vitamin D deficiency during pregnancy.

Characteristic	Vitamin D Deficiency (*n* = 164)	No Vitamin D Deficiency (*n* = 240)	*p*-Value
Gestational age at delivery (weeks)	38.1 ± 1.8	38.4 ± 1.6	0.103
Weight of newborn (g)	2957 ± 472	3013 ± 436	0.253
Height of Newborn (cm)	47.7 ± 2.9	48.3 ± 2.6	0.252
Gestational age at OGTT	24.4 ± 5.2	24.2 ± 5.3	0.655
Glucose Values during OGTT			
- Fasting (mg/dL)	80.0 ± 10.5	78.6 ± 8.6	0.129
- 1 h (mg/dL)	133.6 ± 39.1	122.9 ± 32.7	0.003
- 2 h (mg/dL)	114.8 ± 28.9	105 ± 26.1	0.001
Vitamin D supplementation 1T or 2T	113 (40.9%)	178 (43.8%)	0.563
Vitamin D supplementation doses (IU)	250 (250–500) *	250 (250–500) *	0.165 **
Aspirin started before 17 weeks of gestation	51 (31.1%)	63 (26.3%)	0.226
Metformin started during 1T or 2T	20 (12.2%)	16 (6.6%)	0.055

OGTT = oral glucose tolerance test; 1T = first trimester; 2T = second trimester; * median (interquartile range); ** Mann–Whitney U test.

**Table 3:** Risk of adverse perinatal outcomes in women with and without first-trimester vitamin D deficiency.

Adverse Perinatal Outcome	Vitamin D Deficiency (*n* = 164)	No Vitamin D Deficiency (*n* = 240)	aOR * (95% CI)	*p*-Value
Miscarriage	1 (0.6%)	7 (2.9%)	0.19 (0.02–1.63)	0.09
Gestational diabetes mellitus	34 (20.7%)	24 (10.0%)	2.04 (1.14–3.65)	0.01
Preterm birth	23 (14.0%)	22 (9.2%)	1.44 (0.76–2.52)	0.25
Preeclampsia	20 (12.1%)	18 (7.5%)	1.55 (0.77–3.11)	0.21
Gestational hypertension	4 (2.4%)	13 (5.4%)	0.36 (0.11–1.17)	0.14
Cesarean delivery	91 (55.48%)	137 (57.1%)	0.83 (0.55–1.26)	0.39
Neonates large for gestational age	8 (4.9%)	9 (3.8%)	1.11 (0.41–3.0)	0.84
Neonates small for gestational age	14 (8.5%)	16 (6.7%)	1.27 (0.59–2.7)	0.53

* aOR = Adjusted odds ratio for pre-pregnancy body mass index and obesity; CI = Confidence interval.

## Data Availability

The datasets generated and analyzed during the present study are not publicly available due to ethical restrictions, but are available from the corresponding author on reasonable request.

## Abbreviations

The following abbreviations are used in this manuscript:

APOs	Adverse Perinatal Outcomes
GDM	Gestational Diabetes Mellitus
OBESO	Biochemical and Epigenetic Origin of Overweight and Obesity
OGTT	Oral Glucose Tolerance Test
SGA	Small For Gestational Age
LGA	Large For Gestational Age
BMI	Body Mass Index
